# Adverse kidney outcomes of CDK 4/6 inhibitors for metastatic breast cancer

**DOI:** 10.1038/s41523-023-00576-5

**Published:** 2023-08-19

**Authors:** Paul E. Hanna, Ian A. Strohbehn, Daiana Moreno, Destiny Harden, Rituvanthikaa Seethapathy, Rani Sawtell, Qiyu Wang, Tianqi Ouyang, Nurit Katz-Agranov, James Dinulos, Seth A. Wander, Shruti Gupta, Meghan E. Sise

**Affiliations:** 1 Massachusetts General Hospital, Department of Medicine, Division of Nephrology, Boston, MA USA; 2 Massachusetts General Hospital, Department of Medicine, Division of Hematology and Oncology, Boston, MA USA; 3 Brigham and Women’s Hospital Department of Medicine, Division of Renal Medicine, Boston, MA USA; 4 Adult Survivorship Program, Dana-Farber Cancer Institute, Boston, MA USA

**Keywords:** Breast cancer, Risk factors, Breast cancer, Metastasis

## Abstract

Cyclin-dependent kinase (CDK) 4/6 inhibitors have significantly improved overall and progression free survival of patients with metastatic breast cancer, but their effect on short and long-term kidney function is unknown. We found that early, mild estimated glomerular filtration rate (eGFR) decline was common in patients treated with CDK 4/6 inhibitors; however, severe kidney injury is rare and long-term eGFR decline is uncommon.

Combination of a cyclin-dependent kinase (CDK) 4/6 inhibitor with an aromatase inhibitor is the first-line treatment for patients with hormone receptor (HR)-positive, human epidermal growth factor receptor 2 (HER2)-negative metastatic breast cancer^1^. Previous reports suggest that CDK 4/6 inhibitors may interfere with creatinine secretion in the proximal tubule manifesting with 20–30% decline in estimated glomerular filtration rate (eGFR) without causing true acute kidney injury (AKI)^2–4^. However, a recent case series suggested that CDK 4/6 inhibitors could be associated with intrinsic AKI^5^. Given the contrasting and limited existing evidence, we designed a retrospective cohort study to characterize the short and long-term kidney outcomes in women treated with CDK 4/6 inhibitors and aromatase inhibitors compared to historical controls who received aromatase inhibitors alone^6,7^.

## Methods

We identified a cohort of female patients age ≥18 years with metastatic breast cancer who were treated with CDK4/6 inhibitors (abemaciclib, palbociclib, and ribociclib) and aromatase inhibitors between January 2015 and February 2022, and a cohort of historical controls with metastatic breast cancer treated with aromatase inhibitors alone (anastrozole, letrozole, or exemestane) between September 2007 and December 2014 (prior to the approval of CDK4/6 inhibitors). Baseline eGFR was determined using the most proximal creatinine value within 6 months prior to treatment initiation^8^. Patients without baseline creatinine or without at least one follow-up creatinine within 30 days of starting therapy were excluded. Patients were followed for 12 months.

Patient demographics, laboratory studies, medications, and comorbidities were collected using the Research Patient Data Registry, Mass General Brigham’s centralized clinical data registry. Medication start date and stop date were confirmed by chart review; patients without clear documentation were excluded. Comorbidities and baseline medication use were defined using diagnosis and medication codes prior to the medication start date.

Early 20% eGFR decline was defined by the occurrence of any eGFR measurement 20% below baseline eGFR within the first 30 days after treatment initiation. We also evaluated change in blood urea nitrogen (BUN) and incidence of hematuria and proteinuria within 30 days. Composite adverse kidney outcome was defined by occurrence of >40% decline in eGFR sustained for 90 days, eGFR <10 mL/min/1.73 m^2^ sustained for 90 days, or need for kidney replacement therapy any time within the first year after medication initiation. In the subset of patients who stopped CDK4/6 inhibitor therapy prior to 12 months, we calculated the mean rise in eGFR after discontinuation, comparing the eGFR just prior to discontinuation to the highest eGFR within 30 days.

Baseline characteristics of the cohort were summarized by counts and percentages, or means and standard deviations (SD) for normally distributed data, and median and interquartile range (IQR) for skewed data. A multivariate logistic regression model was used to examine the risk of early 20% eGFR decline among patients receiving abemaciclib or palbociclib vs. aromatase inhibitors alone (reference group). Wald test was used for estimates of the coefficients in logistic regression models and F test was used for ANOVA test. We plotted the mean monthly eGFR calculated using the average of all eGFR measurements within the preceding month for the first 12 months of therapy. Using analysis of variance test, we compared eGFR at 12 months (defined as the average of all eGFR measurements obtained between 11 and 12 months) among patients receiving abemaciclib, palbociclib, or aromatase inhibitors. Ribociclib recipients were excluded in the regression model and eGFR analysis due to low numbers (*N* = 10). Our primary analysis follows the principle of intention to treat and evaluated 1-year eGFR among all patients who began therapy, a secondary analysis evaluated eGFR only among those who remained on therapy for the full 12 months. All analyses were performed using R 4.1.1 and SAS 9.4. The Mass General Brigham Institutional Review Board approved this study and waived the need for informed consent.

**Fig. 1:**
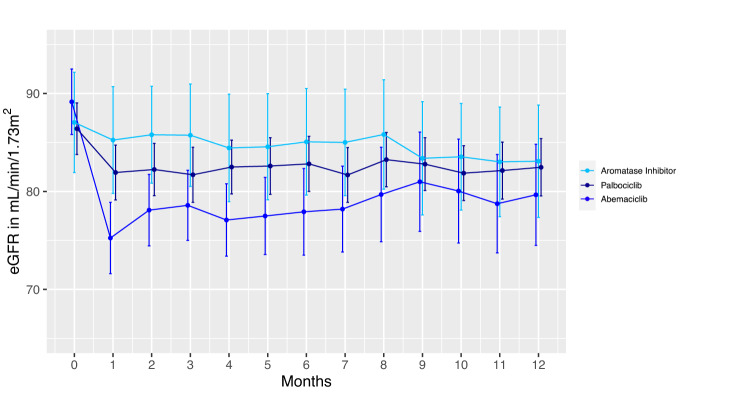
Mean monthly eGFR for the first year of treatment in patients receiving CDK4/6 inhibitors vs. aromatase inhibitors alone. Average eGFR by month and 95% confidence interval bars are shown. There was no significant difference between mean eGFR at month 12 among recipients of abemaciclib (*N* = 67), palbociclib (*N* = 201), or aromatase inhibitors alone (*N* = 63); one-way ANOVA test *p* = 0.59. Ribociclib-treated patients were excluded from this analysis due to low numbers, only 6 survived to 12 months; shown in Supplemental Fig. 2. eGFR estimated glomerular filtration rate.

After applying the exclusion criteria in Supplemental Fig. 1, 474 women with metastatic breast cancer were included; mean age was 60 years (SD 13) and median baseline eGFR was 91 mL/min per 1.73 m^2^ (interquartile range 75–103 mL/min per 1.73 m^2^). Patient characteristics by medication are shown in Table 1. The rate of early 20% eGFR decline was 61% (94/153) in abemaciclib recipients, 22% (52/238) in palbociclib recipients, and 20% (2/10) in ribociclib recipients, compared to 12% (9/73) in patients receiving aromatase inhibitors alone. The risk for early 20% eGFR decline was significantly higher in patients who received abemaciclib (adjusted odds ratio 10.5 [95% CI: 5.0–24, *p* < 0.001]) (Supplemental Table 1). Despite a higher risk of early eGFR decline in abemaciclib recipients, the intention to treat analysis showed that the average eGFR was stable over the next 11 months without significant difference in average eGFR at 12 months among the three groups (Fig. 1; Ribociclib shown in Supplemental Fig. 2). However, patients who remained on abemaciclib for the full 12 months of therapy had a lower eGFR at 12 months, suggesting ongoing inhibition of creatinine secretion (Supplemental Fig. 3). Patients who stopped abemaciclib had the largest rise in eGFR after discontinuation (Supplemental Fig. 4). Despite this, no abemaciclib-treated patients experienced the composite adverse kidney outcome within 12 months. Only 1 patient who received palbociclib experienced sustained >40% eGFR decline; her creatinine returned to baseline after drug discontinuation. No patient developed eGFR <10 mL/min/1.73 m^2^ sustained for 90 days or required kidney replacement therapy at any time within the first year after medication initiation. Changes in BUN, hematuria, and proteinuria within 30 days were not significantly different between treatment groups (Supplemental Table 2).

**Table 1:** Baseline characteristics.

Covariates	Abemaciclib, *N* = 153	Palbociclib, *N* = 238	Ribociclib, *N* = 10	Aromatase inhibitors, *N* = 73
Age	59 (13)	62 (13)	53 (9)	59 (14.49)
Female sex	153 (100%)	238 (100%)	10 (100%)	73 (100%)
Race				
White	130 (85.0%)	199 (83.6%)	9 (90%)	67 (91.8%)
Asian	7 (4.6%)	12 (5.0%)	0 (0%)	1 (1.4%)
Black	8 (5.2%)	10 (4.2%)	0 (0%)	2 (2.7%)
Hispanic	0 (0%)	2 (0.8%)	0 (0%)	0 (0%)
Other/not recorded	8 (5.2%)	15 (6.3%)	1 (10%)	3 (4.1%)
Median baseline eGFR (mL/min/1.73 m^2^)	93 [77, 105]	90 [74, 101]	98 [92, 107]	88 [74, 104]
Comorbidities				
Hypertension	72 (47.1%)	126 (52.9%)	5 (50%)	18 (24.7%)
Coronary artery disease	23 (15.0%)	34 (14.3%)	1 (10%)	4 (5.5%)
Diabetes mellitus	31 (20.3%)	41 (17.2%)	1 (10%)	8 (11.0%)
Cirrhosis	3 (2.0%)	1 (0.4%)	0 (0%)	1 (1.4%)
Medication use				
Diuretics	48 (31.4%)	64 (26.9%)	2 (20%)	12 (16.4%)
ACEi/ARB	32 (20.9%)	63 (26.5%)	1 (10%)	8 (11.0%)
Proton Pump Inhibitors	74 (48.4%)	93 (39.1%)	4 (40%)	14 (19.2%)

Count and percent, or mean and standard deviations, or median and interquartile ranges are shown.

*eGFR* estimated glomerular filtration rate, *ACEi/ARB* Angiotensin Converting Enzyme Inhibitor/Angiotensin Receptor Blocker.

Prior clinical reports provided conflicting evidence on the effect of CKD 4/6 inhibitors on kidney function^2,5^. Wilson et al. reported that 28% of ribociclib-treated patients (9/32) experienced a 50% rise in creatinine; the majority normalized while on the drug or after cessation^9^. Two case reports demonstrated that cystatin C provided a more accurate assessment of kidney function in patients receiving CDK4/6 inhibitors who developed creatinine elevation^2,3^ In contrast, Gupta et al. described a multi-center case series of AKI attributed to CDK 4/6 inhibitors who underwent kidney biopsy showing acute tubular necrosis (*N* = 5) or acute tubulointerstitial nephritis (*N* = 1).

In our study examining a large cohort of women with metastatic breast cancer (*N* = 474), we found an elevated risk of early eGFR decline with abemaciclib, compared to palbociclib and aromatase inhibitor monotherapy. This may be due to abemaciclib’s unique pharmacodynamic and pharmacokinetic features, the fact that it is dosed continually without 1-week pause, or the higher incidence of diarrhea as a side effect^10^. In the unadjusted analyses, we found that baseline variables including hypertension, coronary artery disease, proton pump inhibitor, and diuretics use were associated with 20% eGFR decline within 30 days, however, in the adjusted analyses only abemaciclib use was significantly associated with 20% eGFR decline. Fortunately, severe AKI and the composite adverse kidney outcome were uncommon in patients receiving CDK4/6 inhibitors.

A prior study demonstrated that abemaciclib inhibits organic cation transporter 2 and the multidrug and toxin extrusion proteins in human embryonic kidney cells in vitro, and that despite a rise in serum creatinine in adults treated with abemaciclib, there was no reduction in measured GFR^4^. Despite higher rates of early 20% eGFR decline in patients receiving abemaciclib, we did not detect increased rates of 20% rise in BUN or urinary abnormalities within 30 days. In clinical practice, measurement of cystatin C-based eGFR, which is not affected by medications that inhibit creatinine secretion, may help distinguish between nephrotoxicity and “pseudo-AKI.” Accurate assessment of eGFR is particularly important in light of the approval of abemaciclib in high-risk early-stage disease.

Our study is limited by retrospective design that prohibits causal relationships and are susceptible to unmeasured confounding. The number of ribociclib recipients is limited in our study; additional studies are needed to fully understand kidney risks associated with ribociclib. Future, prospective studies should assess using cystatin C to estimate GFR in patients who receive CKD4/6 inhibitors so that these potentially beneficial therapies are not inappropriately withheld.

### Reporting summary

Further information on research design is available in the Nature Research Reporting Summary linked to this article.

### Supplementary information

Supplementary Materials

Reporting summary

## Data Availability

The data that support the findings of this study are available from the Research Patient Data Registry at Mass General Brigham but restrictions apply as they were used under license for the current study, and so are not publicly available. Data are, however, available from the authors upon reasonable request and with permission from Mass General Brigham.

## Code availability

The R studio code to analyze the data can be made available, however, from the authors upon reasonable request and with permission from Mass General Brigham.
