# Supplementary material for: Adverse kidney outcomes of CDK 4/6 inhibitors for metastatic breast cancer
Source: NPJ Breast Cancer. 2023 Aug 19;9:70. doi: 10.1038/s41523-023-00576-5 (PMC10439887; doi:10.1038/s41523-023-00576-5)
Supplement: Supplementary file 1 — Supplementary Materials [file 41523_2023_576_MOESM1_ESM.docx]

**Supplementary Material**

**TABLE OF CONTENTS**

Supplementary table 1 ………………………………………………………………………….. 2

Supplementary table 2 ………………………………………………………………………….. 3

Supplementary figure 1 ………………………………………………………………………….4

Supplementary figure 2 ………………………………………………………………………….5

Supplementary figure 3 ………………………………………………………………………….6

Supplementary figure 4 ………………………………………………………………………….7

**Supplementary table 1. Predictors of 20% eGFR decline among CDK4/6 inhibitors recipients vs. aromatase inhibitors recipients.**

| Characteristic | Univariable Model | | | Multivariable Model | | |
| --- | --- | --- | --- | --- | --- | --- |
|  | **OR** | **95% CI** | **P-value** | **aOR** | **95% CI** | **P-value** |
| Age | 1.00 | 0.99, 1.02 | 0.93 | 1.00 | 0.98, 1.01 | 0.77 |
| White Race | 1.24 | 0.72, 2.22 | 0.45 | 1.44 | 0.78, 2.75 | 0.25 |
| Baseline eGFR | 1.00 | 0.99, 1.00 | 0.35 |  |  |  |
| Breast cancer therapy |  |  |  |  |  |  |
| Aromatase  Inhibitors | **REF** | **--** | **--** | **REF** | **--** | **--** |
| Abemaciclib | 11.30 | 5.48, 26.0 | **<0.001** | 10.50 | 4.99, 24.4 | **<0.001** |
| Palbociclib | 1.99 | 0.97, 4.52 | 0.08 | 1.83 | 0.87, 4.23 | 0.13 |
| Comorbid Conditions |  |  |  |  |  |  |
| Hypertension | 1.47 | 1.00, 2.16 | **0.05** | 1.16 | 0.68, 1.97 | 0.59 |
| Diabetes  Mellitus | 1.41 | 0.86, 2.31 | 0.17 |  |  |  |
| CAD | 1.71 | 0.98, 2.94 | 0.06 | 1.40 | 0.74, 2.63 | 0.30 |
| Cirrhosis | 3.03 | 0.50, 23.2 | 0.23 |  |  |  |
| Medication Use |  |  |  |  |  |  |
| Proton Pump Inhibitors | 1.59 | 1.07, 2.36 | **0.02** | 1.06 | 0.67, 1.67 | 0.81 |
| Diuretics | 1.83 | 1.19, 2.79 | **0.005** | 1.50 | 0.84, 2.69 | 0.17 |
| ACEi/ARB | 1.29 | 0.81, 2.02 | 0.28 |  |  |  |

**Supplementary table 1.** Logistic regression model for 20% eGFR decline. Ribociclib recipients were excluded from the model due to low numbers (N = 10). Abbreviations: eGFR = estimated Glomerular Filtration Rate, CAD = coronary artery disease ACEi/ARB = Angiotensin Converting Enzyme Inhibitor/Angiotensin Receptor Blocker, OR = odds ratio, aOR = adjusted odds ratio. Wald test was used for estimates of the coefficients in logistic regression models.

**Supplementary table 2. Blood urea nitrogen and urine studies within 30 days of initiating therapy**

|  | Abemaciclib | Palbociclib | Ribociclib | Aromatase Inhibitors |
| --- | --- | --- | --- | --- |
|  | **N=153** | **N=238** | **N=10** | **N=73** |
| 20% increase in Blood Urea Nitrogen |  |  |  |  |
| Present | 53 (35%) | 73 (31%) | 2 (20%) | 24 (33%) |
| Absent | 100 (65%) | 164 (69%) | 8 (80%) | 49 (67%) |
| Missing data (N) | 0 | 1 | 0 | 0 |
| Urine studies: |  |  |  |  |
| Hematuria |  |  |  |  |
| Present | 3 (13%) | 7 (33%) | 2 (40%) | 7 (58%) |
| Absent | 20 (87%) | 14 (67%) | 3 (60%) | 5 (42%) |
| Missing data (N) | 130 | 217 | 5 | 61 |
| Leukocyturia |  |  |  |  |
| Present | 15 (83%) | 10 (67%) | 4 (80%) | 8 (73%) |
| Absent | 3 (17%) | 5 (33%) | 1 (20%) | 3 (27%) |
| Missing data (N) | 135 | 223 | 5 | 62 |
| Proteinuria |  |  |  |  |
| Present | 6 (26%) | 2 (10%) | 1 (20%) | 1 (9%) |
| Absent | 17 (74%) | 19 (90%) | 4 (80%) | 10 (91%) |
| Missing data (N) | 130 | 217 | 5 | 62 |

**Supplementary table 2.** There were no differences in the rate of 20% increase in blood urea nitrogen in patients receiving CDK4/6 inhibitors compared to aromatase inhibitors alone. Conclusions from urinary studies are limited due to the high degree of data missingness and high incidence of concomitant leukocyturia suggesting possible urinary tract infection in patients with urinalyses checked.

**Supplementary figure 1. Patient Flow**

**Supplementary figure 1.** Patient flow showing inclusion and exclusion criteria of patients receiving CDK4/6 and aromatase inhibitors.

**Supplementary figure 2. Mean monthly eGFR values among patients receiving ribociclib**

**Supplementary figure 2.** There were 10 patients who began ribociclib and only 6 survived to 12 months. Monthly mean and 95% confidence interval bars are shown. Abbreviations: eGFR = estimated glomerular filtration rate.

**Supplementary figure 3. Mean monthly eGFR for the first year of treatment in patients receiving CDK4/6 inhibitors vs. aromatase inhibitors alone who continued therapy for the full 12 months**

**Supplementary figure 3**. eGFR trends and 95% confidence interval bars for the patients who completed at least 12 months of therapy are shown above (N = 58 for abemaciclib, N = 133 for palbociclib, and 42 for aromatase inhibitors). Among those remaining on therapy, eGFR remained persistently lower in patients receiving abemaciclib suggesting a persistent effect on creatinine secretion; one-way ANOVA test of mean eGFR at month 12 (p=0.015).

Reasons for early discontinuation of abemaciclib (total N = 153) were disease progression or death in 60 (39%), 32 (21%) stopped due to side effects, and 3 (2%) discontinued therapy for unknown reasons. In the palbociclib group (total N = 238), 66 (28%) stopped due to disease progression or death, 25 (11%) stopped due to side effects, and 14 (6%) discontinued therapy for unknown reasons. Among patients receiving ribociclib (data not shown, total N = 10), 4 (40%) discontinued due to disease progression or death, and 2 (20%) stopped due to side effects. Reasons for early discontinuation of aromatase inhibitors (total N = 73) were disease progression or death in 26 (36%) and 5 (7%) stopped due to side effects.

**Supplementary figure 4. Mean eGFR change within 30 days of starting and stopping therapy.**

**Supplementary figure 4**. Mean change in kidney function in the first 30 days after beginning therapy was defined using the baseline eGFR (just prior to initiation of the CDK 4/6 inhibitor) compared to the lowest eGFR within 30 days of treatment initiation. The increase in eGFR after discontinuation defined as the difference between the eGFR just prior to stopping therapy to the highest eGFR within the 30 days after treatment discontinuation. A total of 95 patients discontinued abemaciclib prior to 12 months and 51 had repeat creatinine within 30 days. A total of 105 patients discontinued palbociclib prior to 12 months and 66 had repeat creatinine within 30 days. A total of 6 patients discontinued ribociclib prior to 12 months and 4 had repeat creatinine within 30 days.
